# Cancer resistance of SR/CR mice in the genetic knockout backgrounds of leukocyte effector mechanisms: determinations for functional requirements

**DOI:** 10.1186/1471-2407-10-121

**Published:** 2010-03-31

**Authors:** Anne M Sanders, John R Stehle, Michael J Blanks, Gregory Riedlinger, Jung W Kim-Shapiro, Arta M Monjazeb, Jonathan M Adams, Mark C Willingham, Zheng Cui

**Affiliations:** 1 Department of Pathology, Wake Forest University School of Medicine Winston-Salem, North Carolina, USA; 2 Molecular Genetics & Genomics Program, Wake Forest University School of Medicine Winston-Salem, North Carolina, USA; 3 Department of Cancer Biology, Wake Forest University School of Medicine Winston-Salem, North Carolina, USA; 4 Radiation Oncology, Wake Forest University School of Medicine Winston-Salem, North Carolina, USA

## Abstract

**Background:**

Spontaneous Regression/Complete Resistant (SR/CR) mice are a colony of cancer-resistant mice that can detect and rapidly destroy malignant cells with innate cellular immunity, predominately mediated by granulocytes. Our previous studies suggest that several effector mechanisms, such as perforin, granzymes, or complements, may be involved in the killing of cancer cells. However, none of these effector mechanisms is known as critical for granulocytes. Additionally, it is unclear which effector mechanisms are required for the cancer killing activity of specific leukocyte populations and the survival of SR/CR mice against the challenges of lethal cancer cells. We hypothesized that if any of these effector mechanisms was required for the resistance to cancer cells, its functional knockout in SR/CR mice should render them sensitive to cancer challenges. This was tested by cross breeding SR/CR mice into the individual genetic knockout backgrounds of perforin (Prf^-/-^), superoxide (Cybb^-/^), or inducible nitric oxide (Nos2^-/^).

**Methods:**

SR/CR mice were bred into individual Prf^-/-^, Cybb^-/-^, or Nos2^-/- ^genetic backgrounds and then challenged with sarcoma 180 (S180). Their overall survival was compared to controls. The cancer killing efficiency of purified populations of macrophages and neutrophils from these immunodeficient mice was also examined.

**Results:**

When these genetically engineered mice were challenged with cancer cells, the knockout backgrounds of Prf^-/-^, Cybb^-/-^, or Nos2^-/- ^did not completely abolish the SR/CR cancer resistant phenotype. However, the Nos2^-/- ^background did appear to weaken the resistance. Incidentally, it was also observed that the male mice in these immunocompromised backgrounds tended to be less cancer-resistant than SR/CR controls.

**Conclusion:**

Despite the previously known roles of perforin, superoxide or nitric oxide in the effector mechanisms of innate immune responses, these effector mechanisms were not required for cancer-resistance in SR/CR mice. The resistance was functional when any one of these effector mechanisms was completely absent, except some noticeably reduced penetrance, but not abolishment, of the phenotype in the male background in comparison to female background. These results also indicate that some other effector mechanism(s) of granulocytes may be involved in the killing of cancer cells in SR/CR mice.

## Background

Spontaneous Regression/Complete Resistant (SR/CR) mice are a mouse model that is capable of resisting lethal challenges with a wide variety of cancers [1,2]. The resistance resides primarily in innate leukocytes consisting of granulocytes, monocytes, and natural killer cells which migrate to the site of the tumor, recognize the cancer cells via tight contact, and then destroy the tumor cells mainly through cytolysis [1,2]. The cytolysis of cancer cells in SR/CR mice was previously indicated to involve multiple effector mechanisms [1-3], a number of which are associated with innate immunity. Specifically, perforin and granzymes were detected in the peritoneal fluid and in a fraction of the rosettes following a challenge with S180, in addition to a decrease in S180 killing by SR/CR macrophages following the inhibition of reactive oxygen species [3].

Several unanswered questions remain pertaining to the roles that these various effector mechanisms play in the killing of cancer cells by SR/CR leukocytes. In particular, it is unknown what proportion of the perforin positive cells were natural killer (NK) cells or cytotoxic T lymphocytes (CTL) and therefore the significance of perforin in the primary response and its role in the NK killing activity in these mice remain unclear. Furthermore, since inhibitors can have nonspecific effects on other enzymes or can have incomplete inhibition, it is not completely clear if the superoxide and nitric oxide effector mechanisms are required for the SR/CR cancer resistance phenotype, or for the cancer killing activity of an individual leukocyte population. In order to thoroughly evaluate these effector mechanisms during the primary anticancer response in a system with complete and specific inhibition, SR/CR mice were bred into genetic backgrounds deficient in perforin, superoxide, and nitric oxide.

Perforin is a protein found in secretory vesicles of CTL and NK cells, encoded by its gene on chromosome 10 [4]. When released at the immunologic synapse between a leukocyte and its target, perforin polymerizes and forms pores in the target's membranes [5]. While perforin is an inefficient cytolytic agent by itself; it facilitates the release of granzymes into the cytosol of the target cells and then triggers apoptotic pathways [6]. In the perforin knockout mice (Prf^-/-^), CTL and NK cells are present in normal numbers, but are unable to lyse virus-infected or allogeneic fibroblasts *in vitro *[7]. Perforin knockout mice are also more susceptible to viral pathogens, spontaneous B cell lymphomas, and transplanted or inducible tumors [7-11]. In the primary SR/CR response to cancer, it is likely that the absence of perforin would have the greatest effect on NK cells, since CTL's would require an initial priming event.

Superoxide is one type of reactive oxygen species that is produced by both macrophages and neutrophils for host defense. At the immunologic synapse or phagosome, superoxide is generated by NADPH oxidase. NADPH oxidase is a multi-subunit complex that catalyzes the reduction of molecular oxygen at the expense of NADPH [12]. The gp91^phox ^subunit, also known as NOX2, is an essential membrane bound protein that helps form the redox center of the enzyme [13-15]. If the gp91^phox ^subunit is missing or defective, NADPH oxidase is inactive [16-18]. Mice with null alleles for gp91^phox ^(Cybb^-/-^) on the X chromosome lack phagocyte superoxide production making them more susceptible to some bacterial and fungal infections [16]. The absence of superoxide would likely affect both macrophages and neutrophils in the primary SR/CR response to cancer; and with twice the capacity for reactive oxygen species production, neutrophils may be affected the greatest [19].

Macrophages and neutrophils also produce nitric oxide, a reactive nitrogen species that is somewhat complementary to superoxide [20]. At the immunologic synapse or phagosome, nitric oxide is generated by inducible nitric oxide synthase (Nos2) which converts arginine to citruline and nitric oxide, using molecular oxygen and NADPH [21,22]. Mice with null Nos2 alleles on chromosome 11 (Nos2^-/-^) have virtually no serum nitric oxide response to lipopolysaccharide (LPS) [23] and have an altered response to many infections [24] and impaired wound healing [25]. Since Nos2 inhibitors were previously shown not to have an effect on macrophage killing *in vitro *[3], it is likely that the absence of nitric oxide will only affect neutrophil killing activity during the primary SR/CR response to cancer *in vivo*.

Here, we report the findings of the SR/CR mice bred into the knockout backgrounds of Prf-/-, Cybb-/-, and Nos2-/- as it relates to the survival against a primary challenge with S180, and the evaluation of the cancer killing activity of individual leukocyte populations from these mice.

## Methods

### Cell Lines and Mouse Strains

The S180 cell line was obtained from the ATCC (Manassas, VA). S180 cells were either propagated in DMEM with 10% FBS at 37°C in 5% carbon dioxide or maintained by serial passages through wild-type (WT) C57BL/6 mice as cancerous ascites. WT C57BL/6J mice, C57BL/6-RAG1^tm1Mom ^mice, B6.129S6-Cybb^tm1Din^/J, B6.129P2-Nos2^tm1Lau^/J, and C57BL/6-Prf1^tm1Sdz^/J mice were purchased from The Jackson Laboratory (Bar Harbor, ME). SR/CR mice in the C57BL/6 congenic background were bred at the ARP facility of WFU [1]. Animals were housed under 12-hour light/dark cycles and received a standard laboratory chow diet. All protocols and procedures were approved by the IACUC of the WFU Health Sciences.

### Prf Breeding and Screening

SR/CR C57BL/6 mice were bred with WT Prf^-/- ^mice. At six weeks of age, the first filial generation (F1) progeny were screened with 1 × 10e6 S180 i.p., then 5 × 10e6 at ten weeks. Survivors were considered resistant, or F1 SR/CR Prf^+/- ^mice, and used to breed the backcross (BC or F2) generation. F1 SR/CR Prf^+/- ^were bred with WT Prf^-/- ^mice. All of the BC progeny were Prf PCR genotyped at 3-4 weeks of age. At 6 weeks of age, the BC progeny were screened with 1 × 10e6 S180 i.p., then 5 × 10e6 at ten weeks. The second challenge of 5 × 10e6 was instituted as a safeguard against occasional failed first injections. All WT control mice were succumbed to the challenges via cancerous ascites. Healthy survivors were considered resistant, or BC SR/CR mice.

### Prf PCR

Tail snips were digested in DirectPCR Lysis Reagent-Tail (Viagen), with 0.4 mg/mL proteinase K. One uL of the lysate was used for each PCR reaction. Prf PCR genotyping was performed according to The Jackson Laboratory standard procedures by simultaneous amplification of the WT (Prf^+^) and knockout (Prf^-^) alleles using three primers: oIMR1100, 5'-GCTATCAGGACATAGCGTTGG-3'; oIMR3108, 5'-GGAGGCTCTGAGACAGGCTA-3'; and oIMR3109, 5'-TACCACCAAATGGGCCAAG-3'. The PCR yielded products of sizes 187 bp Prf^+ ^and 250 bp Prf^-^, which were analyzed on 2% agarose gels.

### Cybb Breeding and Screening

SR/CR C57BL/6 female mice were bred with WT Cybb^Y/- ^male mice. At six weeks of age, the F1 progeny were screened with 1 × 10e6 S180 i.p., then 5 × 10e6 at ten weeks. Male survivors were designated F1 SR/CR Cybb^Y/+^. SR/CR female survivors were designated F1 SR/CR Cybb^+/-^, and used to breed the BC generation. F1 SR/CR female Cybb^+/- ^were bred with WT Cybb^Y/- ^male mice. All of the BC progeny were Cybb PCR genotyped at 3-4 weeks of age. At 6 weeks of age, the BC progeny were screened with 1 × 10e6 S180 i.p., then 5 × 10e6 at ten weeks. Survivors were considered resistant, or BC SR/CR mice.

### Cybb PCR

Tail snips were digested in DirectPCR Lysis Reagent-Tail (Viagen), with 0.4 mg/mL proteinase K. 1 uL of the lysate was used for each PCR reaction. Cybb PCR genotyping was performed according to The Jackson Laboratory standard procedures by simultaneous amplification of the WT (Cybb^+^) and knockout (Cybb^-^) alleles using three primers: oIMR0517, 5'-AAGAGAAACTCCTCTGCTGTGAA-3'; oIMR0518, 5'-CGCACTGGAACCCCTGAGAAAGG-3'; and oIMR0519, 5'-GTTCTAATTCCATCAGAAGCTTATCG-3'. The PCR yielded products of sizes 240 bp Cybb^+ ^and 195 bp Cybb^-^, which were analyzed on 2% agarose gels.

### Histology

When showing signs of expected illness, the mice were sacrificed. Their tissues were fixed in 10% neutral buffered formalin, and subsequently embedded in paraffin. Sections were stained with hematoxylin and eosin and then examined.

### Nos2 Breeding and Screening

SR/CR C57BL/6 mice were bred with WT Nos2^-/- ^mice. At six weeks of age, the F1 progeny were screened with 1 × 10e6 S180 i.p., then 5 × 10e6 at ten weeks. Survivors were considered resistant, or F1 SR/CR Nos2^+/- ^mice, and used to breed the BC generation. F1 SR/CR Nos2^+/- ^were bred with WT Nos2^-/- ^mice. All of the BC progeny were Nos2 PCR genotyped at 3-4 weeks of age. At six weeks of age, the BC progeny were screened with 1 × 10e6 S180 i.p., then 5 × 10e6 at ten weeks. Survivors were considered resistant, or BC SR/CR mice.

### Nos2 PCR

DNA was isolated from tail snips using the Wizard Genomic DNA Purification Kit (Promega, A1120). Nos2 PCR genotyping was performed according to The Jackson Laboratory standard procedures by simultaneous amplification of the WT (Nos2^+^) and knockout (Nos2^-^) alleles using three primers: oIMR1216, 5'-ACATGCAGAATGAGTACCGG-3'; oIMR1217, 5'-TCAACATCTCCTGGTGGAAC-3'; and oIMR1218, 5'-AATATGCGAAGTGGACCTCG-3'. The PCR yielded products of sizes 108 bp Nos2^+ ^and 270 bp Nos2^-^, which were analyzed on 2% agarose gels.

### Macrophage Isolation

Four days after a 2 mL i.p. injection with 2% thioglycolate (Sigma, B2551), macrophages were harvested by peritoneal lavage. The cells were cultured on tissue culture dishes for 1.5 hours in media (DMEM with 10% FBS) at 37°C. Non-adherent cells were removed by two rinses with phosphate buffered saline (PBS) containing calcium, and discarded. The adherent cells were incubated for 1 hour in 5 mM EDTA (in DMEM with 10% FBS) at 37°C. The macrophages were vigorously washed off using PBS without calcium, counted by Trypan Blue exclusion, and resuspended at the appropriate concentrations. Purity was > 95% macrophages as determined by hematoxylin staining and the unique morphology of macrophages.

### Neutrophil Isolation

Eighteen hours after a 2 mL i.p. injection of 2% thioglycolate, neutrophils were harvested by peritoneal lavage. The cells were cultured for 1 hour in media (DMEM with 10% FBS) at 37°C. Non-adherent cells were collected by several light rinses with PBS containing calcium, counted by Trypan Blue exclusion, and resuspended at the appropriate concentrations. Purity was > 85% neutrophils as determined by hematoxylin staining and typical polymorphic nuclei.

### Griess Assay

In a 96-well plate, 2 × 10e5 macrophages or 4 × 10e5 neutrophils were plated in DMEM with 10% FBS, at a final volume of 200 uL. Ten ug/mL stocks of interferon-gamma (IFNγ; Sigma, I4777) and lipopolysaccaride (LPS; E. Coli Serotype 0111:B4, Fluka, 62325) were freshly mixed in a 1:1 ratio. Four uL of the mixture was added to each stimulated well, while nothing was added to the unstimulated controls. The cells were incubated at 37°C for 24 hours. The following day, 50 uL of the media from each well was transferred to a flat-bottomed 96-well plate. In duplicate, a standard curve was also made with sodium nitrite in media (124-1.9 uM, and media alone; Sigma, S-2252) with a final volume of 50 uL per well. Fifty uL of Griess reagent (Fluka, 03553) was added to each well, and gently mixed. Large bubbles were popped with a heated needle. After 15 minutes at room temperature, the absorbance at 540 nm was read in a plate reader. The nitrite production for each sample was calculated (average stimulated - average unstimulated nitrite production), using the standard curve.

### Adoptive Transfers

RAG1^-/- ^recipients were given 8-10 × 10e6 macrophages or neutrophils i.p. The following day, the recipients were challenged with 1 × 10e4 S180 i.p. and their survival was monitored. Moribund mice were euthanized and examined for the presence of tumor. Mice that were healthy and ascites free, 6 weeks post injection, were considered resistant.

### Statistical Analysis

Statistical analysis was performed by the two-tailed Student's t-test. P-values less than 0.05 were regarded as statistically significant.

## Results and Discussion

### Prf Breeding and Screening

**Figure 1:**
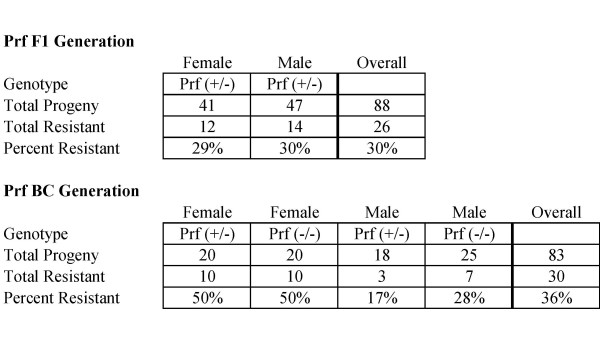
**SR/CR Mice Do Not Require Perforin to Survive S180 Challenges**. SR/CR mice were bred into the perforin (Prf) knockout background. Progeny that survived two challenges with S180, remaining healthy and cancer free, were considered resistant. Similar survival rates were seen for the BC Prf^+/- ^and Prf^-/- ^mice.

SR/CR Prf^+/+ ^mice were bred with WT Prf^-/- ^mice. All F1 progeny were expected to be Prf^+/-^. When challenged with S180, 30% of the progeny survived (Figure 1). This was similar to the survival seen in SR/CR Prf^+/+ ^mice [1] which suggested that the single null allele did not have a major effect on the SR/CR phenotype. The surviving SR/CR F1 Prf^+/- ^mice were fertile and were used to breed the BC generation.

SR/CR F1 Prf^+/- ^mice were crossed with WT Prf^-/- ^mice. As expected, about half of the BC progeny born were Prf^+/- ^and half were Prf^-/- ^(Figure 1), indicating that perforin knockout was not embryonic lethal. When challenged with S180, 36% of the progeny survived, which was similar to the survival rate in the SR/CR F1 Prf^+/- ^mice. However, there was a significant survival bias in the BC generation in which the females were twice as likely to be resistant than the males (p = 0.012). This was unexpected because there was no gender bias observed in the F1 generation, and neither the SR/CR mutation nor perforin have been associated with the sex chromosomes. In contrast, there was no significant difference in overall survival between the Prf^+/- ^and Prf^-/- ^mice, indicating that perforin was not required for the initial SR/CR phenotype *in vivo*.

### Cybb Breeding and Screening

**Figure 2:**
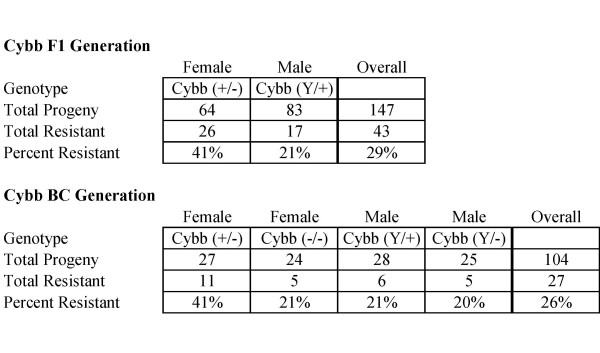
**SR/CR Mice Do Not Require Superoxide to Survive S180 Challenges**. SR/CR mice were bred into the superoxide (Cybb) knockout background. Progeny that survived two challenges with S180, remaining healthy and cancer free, were considered resistant. Similar survival rates were seen for the BC progeny with or without Cybb; however, there was a trend that the males were more cancer sensitive.

One noticeable thing was that the purchased breeders with Cybb-KO were created in a mixed genetic background of C57BL/6 and 129. Nevertheless, the potential impact of a mixed genetic background between C57BL/6 and 129 is currently unknown. SR/CR female Cybb^+/+ ^mice were crossed with WT male Cybb^Y/- ^mice. The males were expected to be normal, or Cybb^Y/+^, and the females were expected to be Cybb^+/-^. When challenged with S180, 29% of the progeny survived (Figure 2), which was similar to the rate seen in SR/CR with normal NADPH oxidase genes [1]. Again, there was a significant female survival bias (p = 0.008). Even though the males should have normal NADPH oxidase activity, the F1 females were twice as likely to be resistant. Overall, the single null allele in the females did not appear to have a significant negative effect on the SR/CR phenotype, so the surviving SR/CR F1 Cybb^+/- ^mice which were fertile were then used to breed the BC generation.

SR/CR F1 female Cybb^+/- ^mice were crossed with WT male Cybb^Y/- ^mice to generate the BC generation. The choice of using female F1s as breeders was to generate all 4 possible groups: SR/Cybb-KO/males, SR/Cybb-KO/females, WT/Cybb-KO/males and WT/Cybb-KO/females. As a result, SR/CR groups can be compared to WT groups that came from a same breeding scheme. There was an even distribution of mice born by gender and expected Cybb genotypes (Figure 2), indicating that there was no embryonic lethality. When challenged with S180, 26% of the progeny survived, which was similar to the survival rate seen in the F1 mice. There were no significant differences in survival by either gender or genotype; but there was a general trend that female Cybb^+/- ^mice had a higher frequency of resistance than the other groups. Since there were resistant Cybb^-/- ^females and Cybb^Y/- ^males, superoxide did not appear to be required for the initial SR/CR phenotype *in vivo*.

### Cybb Histology

**Figure 3:**
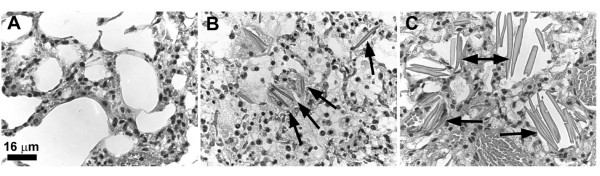
**Lung Crystals Found in Moribund SR/CR Cybb^-/- ^and Cybb^Y/- ^Mice**. Hematoxylin and eosin stained sections of lung showed the presence of large eosinophilic crystals (arrows) in the lungs of moribund Cybb^Y/- ^(B) and Cybb^-/- ^(C) mice. No crystals were observed in the lungs of age-matched SR/CR Cybb^+/- ^(A) or Cybb^+/+ ^mice.

Tissues were taken from older, moribund SR/CR Cybb^-/- ^and Cybb^Y/- ^mice to check for malignancies. Large refractile crystals were observed in the lungs of some of the mice (Figure 3); but were not detected in the lungs of any of the SR/CR Cybb normal or Cybb^+/- ^mice. The majority of the crystals were slender rods with tapered ends; although, a few were larger rhomboids. Following hematoxylin and eosin staining, the crystals were eosinophilic (light pink) and translucent. While one of the mice had a few scattered crystals, the rest had an abundance of crystals associated with inflammatory sites. Macrophages and multinucleated macrophage giant cells were often seen adjacent to the crystals, possibly attempting to engulf them. The crystals were similar in appearance to Charcot-Leyden crystals that consist of lysophospholipase and are associated with pulmonary inflammation [26]. They are also similar in appearance to Ym1 crystals found in the aging lungs and at sites of chronic inflammation in p47^phox ^knockout mice [27]. YM1 is a chitinase family protein that is found in macrophages and myeloid cells located in the spleen and bone marrow and, also, pulmonary macrophages [28]. p47^phox ^knockout mice are phenotypically similar to Cybb^-/- ^mice since they both have defective phagocyte NADPH oxidase complexes and are consequently, superoxide deficient. Therefore, it is highly likely that the crystals are composed of Ym1.

### Nos2 Breeding and Screening

**Figure 4:**
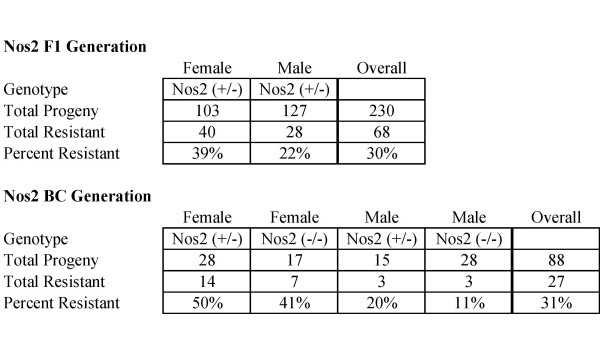
**SR/CR Mice Do Not Require Nitric Oxide to Survive S180 Challenges**. SR/CR mice were bred into the inducible nitric oxide synthase (Nos2) knockout background. Progeny that survived two challenges with S180, remaining healthy and cancer free, were considered resistant. Similar survival rates were seen for the BC Nos2+/- and Nos2-/-; however, there was a strong trend that the males were more cancer sensitive.

SR/CR Nos2^+/+ ^mice were crossed with WT Nos2^-/- ^mice, and all F1 progeny were expected to be Nos2^+/-^. When challenged with S180, 30% of the progeny survived (Figure 4). Since this was similar to the survival rate seen in SR/CR Nos2^+/+ ^mice [1], the single null allele did not appear to have a significant effect on the SR/CR phenotype. However, there was a significant survival bias based on gender (p = 0.006) in which females were about twice as likely to be resistant than males. This was unexpected since neither the SR/CR mutation nor the Nos2 gene has been associated with the sex chromosomes. The surviving SR/CR F1 Nos2^+/- ^mice were fertile, and were used to breed the BC generation.

SR/CR F1 Nos2^+/- ^mice were crossed with WT Nos2^-/- ^mice to generate the BC generation. As expected, about half the BC progeny were Nos2^+/- ^and half were Nos2^-/- ^(Figure 4), indicating that there was no embryonic lethality. When challenged with S180, 31% of the progeny survived, which was similar to the survival seen in the SR/CR F1 Nos2^+/- ^mice. Again, there was a significant survival bias based on gender (p = 0.002), in which females were twice as likely to be resistant compared to males. Furthermore, there was a strong general trend that Nos2^-/- ^mice were less resistant than Nos2^+/- ^mice (p = 0.078). Importantly, this is the first time in which the absence of a single effector mechanism, namely Nos2, may have a negative effect, but not abolishment, on the germline transmission rate of SR/CR anticancer response *in vivo*.

### Nitric Oxide Production

**Figure 5:**
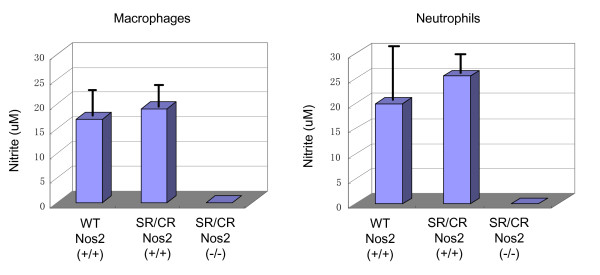
**SR/CR Nos2 Knockout Macrophages and Neutrophils Have No Detectable Nitric Oxide Production Following LPS and IFNγ Stimulation**. Thioglycolate elicited macrophages and neutrophils were stimulated with LPS and IFNγ for 24 hours. Nitrite levels, an indirect measure of nitric oxide production, were assessed by the Griess Assay. Nitrite levels for WT (n = 5) and SR/CR (n = 5) Nos2^+/+ ^macrophages were comparable, but significant levels of nitrites were not detected (n.d.) in SR/CR Nos2^-/- ^macrophages (n = 3). Nitrite levels for WT (n = 4) and SR/CR (n = 3) Nos2^+/+ ^neutrophils were also comparable, but significant levels of nitrites were not detected in SR/CR Nos2^-/- ^neutrophils (n = 3).

In order to confirm that nitric oxide production was absent in SR/CR Nos2^-/- ^macrophages and neutrophils, the Griess assay was performed. Since nitric oxide is a short-lived molecule, one of its more stable by-products, nitrite, is commonly measured. Following stimulation with interferon gamma (IFNγ) and LPS, the nitrite production of macrophages and neutrophils from WT Nos2^+/+^, SR/CR Nos2^+/+^, and SR/CR Nos2^-/- ^mice was quantified (Figure 5). As expected, macrophages and neutrophils from both WT and SR/CR Nos2^+/+ ^mice had robust nitrite production; while SR/CR Nos2^-/- ^macrophages and neutrophils had no detectable nitrite production.

### Adoptive Transfers

**Figure 6:**
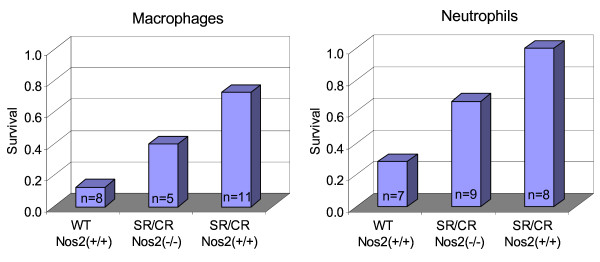
**Adoptive Transfers of SR/CR Nos2 Knockout Macrophages and Neutrophils are Less Protective**. Thioglycolate elicited SR/CR macrophages and neutrophils were adoptively transferred into WT RAG1^-/- ^mice. The WT RAG1^-/- ^recipients were challenged the following day with S180, and their survival was monitored. Survival of SR/CR Nos2^-/- ^recipients was intermediate, indicating that nitric oxide was one of the effector mechanisms used by SR/CR macrophages and neutrophils to kill cancer.

Nitric oxide is one of the known effector mechanisms used by macrophages and neutrophils. If nitric oxide was the chief effector mechanism used by SR/CR macrophages or neutrophils to kill cancer cells, then the absence of nitric oxide would significantly weaken or abolish this activity. The cancer killing activity of SR/CR Nos2^-/- ^macrophages and neutrophils was tested by transferring purified populations of each cell type to a non-resistant WT recipient followed by a challenge with lethal cancer cells. To study the effect of the transferred innate leukocytes in the absence of adaptive immunity, WT recombination activating gene 1 (Rag1) knockout mice were used as recipients. By using this experimental setup, we were able to determine whether SR/CR Nos2^-/- ^macrophages and neutrophils could transfer the anticancer phenotype as efficiently as SR/CR Nos2^+/+ ^macrophages and neutrophils. The same survival trend was seen in both the macrophage and neutrophil recipients in which the SR/CR Nos2^-/- ^leukocytes conferred less protection when compared to SR/CR Nos2^+/+ ^leukocytes (Figure 6). These results suggest that nitric oxide may be one of the major effector mechanisms used by SR/CR macrophages and neutrophils to kill cancer.

## Conclusions

The SR/CR anticancer response was previously shown to involve multiple leukocyte subsets that can work collectively or independently [1-3]. It has also been shown that the anticancer response of the SR/CR mice involves multiple effector mechanisms [3]. However, it was not known if the complete abolishment of any one effector mechanism would affect the SR/CR anticancer response due to the overlapping functional roles that many of the leukocyte populations displayed. Our current study shows that the SR/CR mice were capable of surviving a primary challenge with a lethal dose of S180 even with the genetic knockout of perforin, superoxide, or nitric oxide. In the absence of any one of these effector mechanisms, the cancer cells can still be destroyed leading to survival of the challenged mice. From these results, it is likely that only global immunosuppression, affecting multiple leukocyte populations and their effector mechanisms, would be able to abolish the SR/CR phenotype.

One intriguing finding was the important role that nitric oxide plays in the anticancer activity of the SR/CR macrophages and neutrophils. When SR/CR Nos2^-/- ^macrophages and neutrophils were transferred into cancer-sensitive WT mice, the SR/CR Nos2^-/- ^cells proved to be less protective against an S180 challenge compared to cells from SR/CR Nos2^+/+ ^mice. These results suggest that nitric oxide may be one of the major effector mechanisms used by SR/CR macrophages and neutrophils to kill cancer cells. However, at the same time these results also suggest that the SR/CR macrophages and neutrophils have additional anticancer effector mechanisms in place, since the Nos2^-/- ^transferred cells still provided some protection. In order to continue to evaluate the significance of other effector mechanisms, the killing activity of macrophages and neutrophils from SR/CR Nos2^-/- ^mice will need to be assessed in combination with inhibitors of the other known effector mechanisms. Since the knockouts of perforin, superoxide and nitric oxide alone did not abolish the anticancer ability of the SR/CR mice, then the production of SR/CR mice with multiple effector mechanism knockouts may also be necessary for more complete characterization of the SR/CR phenotype.

The gender bias that was observed among all three knockout lines of mice was quite an unexpected finding. In several generations, female mice displayed a significantly higher percentage of cancer resistance among the challenged mice. Since the SR/CR mutation, Nos2 gene, and perforin gene are not X-linked, it is possible that there may be an unidentified X-linked gene that may enhance the SR/CR phenotype. However, since the gender bias is not consistently observed in the SR/CR breeding (data not shown), the bias may alternatively stem from the knockout backgrounds. The influence that hormone levels play may be another explanation for the differences observed between males and females. Many leukocyte subsets express hormone receptors [29] and in comparison to males, females tend to have more vigorous cellular and humoral immune reactions which are thought to make them more resistant to certain infections and at greater risk for autoimmunity [30]. WT males and females appear equally sensitive to S180; however, when the SR/CR phenotype is in an immunocompromised background, the more vigorous immune response of the females may provide a slight survival advantage. One more explanation for the sex bias could reside in the fact that S180 immunostain positively for a Y-marker antigen (unpublished results), which would make S180 more antigenic in a female background. In males, this would be recognized as a self epitope, while in females it would be recognized as a foreign epitope and, therefore, could promote rejection of the transplanted cancer. The presence of a Y-antigen on S180 does not appear to matter for mice with a cancer-sensitive WT background, since males and females equally succumb to cancer challenge. However, when the SR/CR phenotype is introduced into an immunocompromised background, the extra antigenicity from the Y-antigen could provide a slight survival advantage for female mice.

Another possibility for this sex bias could be due to a possible weakening of the resistance strength as a result of continuous breeding for years. For example, in the first few generations of SR/CR mice, they could tolerate challenges of up to 10e9 S180 cells. In recent years, however, the maximum tolerated dose (MTD) seemed to have dropped to about 10e8 S180 cells (Cui et all, unpublished observations). Even with such a drop in MTD, the resistant phenotype of SR/CR mice is still unequivocal in comparison to non-resistant (WT) mice that would succumb to less than 10e5 or even less than 10e4 S180 cells. The decrease of MTD may reflect a possibility that the genetic components in the early generations may have multiple copies that were reduced to fewer copies or single copy in the later generations after years of breeding. If this is the case, the high MTD in early generations of SR/CR mice might have overcome some subtle influences, such as sex background. On the other hand, some subtle influences may show up when MTD was decreased as a result of fewer copy number for the underlying gene/mutation. The MTD could also be further decreased, but not abolished, by knockout of some participating but essential pathway for effector mechanisms. This could further exacerbate the impact of otherwise subtle influences.

Collectively these findings support the prior reports that the SR/CR anticancer response is the result of a concerted effort from several leukocyte populations that utilize multiple effector mechanisms. The many overlapping processes enable the host to achieve the same end result, elimination the cancer cells and survival. These findings also demonstrate that a highly effective anticancer response can be mediated by innate cellular immunity that requires no additional manipulation, supporting the idea that leukocytes of innate immunity could potentially be used as therapeutic agents to prevent or even to treat cancers.

## Competing interests

The authors declare that they have no competing interests.

## Authors' contributions

AS helped conceive of the study, participated in its design and coordination, helped perform the injections, lavages, adoptive transfers, helped with animal husbandry, and drafted the manuscript. JS helped design the studies, perform the lavages, and with preparation of manuscript and figures. MB helped design the studies, and with manuscript and figure preparation. GR helped design the studies and with manuscript preparation. JK helped with the final analyses and with manuscript preparation. AM helped with the final analyses and with manuscript preparation. JA helped perform the injections, lavages, animal care and helped with manuscript preparation. MW helped design the studies, helped with the histological evaluation, and with manuscript preparation. ZC was responsible for the oversight of the entire project and including experimental design and manuscript writing. All authors read and approved the final manuscript.

## Pre-publication history

The pre-publication history for this paper can be accessed here:

http://www.biomedcentral.com/1471-2407/10/121/prepub
